# Early Postnatal but Not Late Adult Neurogenesis Is Impaired in the Pitx3-Mutant Animal Model of Parkinson's Disease

**DOI:** 10.3389/fnins.2017.00471

**Published:** 2017-08-24

**Authors:** Moritz D. Brandt, Diana Krüger-Gerlach, Andreas Hermann, Anne K. Meyer, Kwang-Soo Kim, Alexander Storch

**Affiliations:** ^1^Department of Neurology, Technische Universität Dresden Dresden, Germany; ^2^German Center for Neurodegenerative Diseases Dresden Dresden, Germany; ^3^Center for Regenerative Therapies Dresden, Technische Universität Dresden Dresden, Germany; ^4^Molecular Neurobiology Laboratory, McLean Hospital/Harvard Medical School Belmont, MA, United States; ^5^German Center for Neurodegenerative Diseases Rostock Rostock, Germany; ^6^Department of Neurology, University of Rostock Rostock, Germany

**Keywords:** adult neurogenesis, Parkinson's disease, Pitx3, aphakia, aging, running

## Abstract

The generation of new neurons in the adult dentate gyrus has functional implications for hippocampal formation. Reduced hippocampal neurogenesis has been described in various animal models of hippocampal dysfunction such as dementia and depression, which are both common non-motor-symptoms of Parkinson's disease (PD). As dopamine plays an important role in regulating precursor cell proliferation, the loss of dopaminergic neurons in the substantia nigra (SN) in PD may be related to the reduced neurogenesis observed in the neurogenic regions of the adult brain: subventricular zone (SVZ) and dentate gyrus (DG). Here we examined adult hippocampal neurogenesis in the Pitx3-mutant mouse model of PD (*aphakia* mice), which phenotypically shows a selective embryonic degeneration of dopamine neurons within the SN and to a smaller extent in the ventral tegmental area (VTA). Proliferating cells were labeled with BrdU in *aphakia* mice and healthy controls from 3 to 42 weeks of age. Three weeks old mutant mice showed an 18% reduction of proliferating cells in the DG and of 26% in the SVZ. Not only proliferation but also the number of new neurons was impaired in young *aphakia* mice resulting in 33% less newborn cells 4 weeks after BrdU-labeling. Remarkably, however, the decline in the number of proliferating cells in the neurogenic regions vanished in older animals (8–42 weeks) indicating that aging masks the effect of dopamine depletion on adult neurogenesis. Region specific reduction in precursor cells proliferation correlated with the extent of dopaminergic degeneration in mesencephalic subregions (VTA and SN), which supports the theory of age- and region-dependent regulatory effects of dopaminergic projections. Physiological stimulation of adult neurogenesis by physical activity (wheel running) almost doubled the number of proliferating cells in the dentate gyrus of 8 weeks old *aphakia* mice to a number comparable to that of wild-type mice, abolishing the slight reduction of baseline neurogenesis at this age. The described age-dependent susceptibility of adult neurogenesis to PD-like dopaminergic degeneration and its responsiveness to physical activity might have implications for the understanding of the pathophysiology and treatment of non-motor symptoms in PD.

## Introduction

Structural change of neuronal networks in the brain is an important mechanism for learning and memory (Shors et al., 2001). Generation and degeneration of synaptic connections as well as the addition of new neurons in the dentate gyrus and olfactory bulb maintaining cell turnover in these regions provide optimal network function via neuronal plasticity. Particularly neurogenesis in the adult brain as a maximal way of cellular plasticity has been investigated in various animal models of neuropsychiatric diseases as well as the human brain. Impaired neurogenesis in the dentate gyrus of the hippocampus has been suggested to play an important role in the pathophysiology of depression and cognitive impairment (Feng et al., 2001; Kempermann and Kronenberg, 2003; Snyder et al., 2011; Hill et al., 2015; Schoenfeld and Cameron, 2015). Taken together, adult neurogenesis is a physiological mechanism with functional relevance for structures that benefit from an increased cell turnover: the dentate gyrus of the hippocampus and the olfactory bulb.

Depression, cognitive impairment and hyposmia are common non-motor symptoms of Parkinson's disease (PD; Reichmann et al., 2016), a neurodegenerative disease that is characterized by the loss of dopaminergic neurons in the mesencephalon. As dopamine plays an important role in regulating neurogenesis during embryonic development (Diaz et al., 1997), it has already been suggested that alterations of dopamine level in PD might also affect adult neurogenesis in the neurogenic regions and thereby promote the development of depression, dementia and hyposmia (Marxreiter et al., 2013b). Hoeglinger et al. were the first to investigate adult neurogenesis after acute degeneration of the mesencephalic dopaminergic system using the neurotoxins MPTP and 6-hydroxydopamine (6-OHDA), respectively (Hoglinger et al., 2004). They observed significant reduction of cell proliferation in the subgranular zone of the dentate gyrus (SGZ) and the subventricular zone (SVZ), a region containing stem- and precursor cells that give rise to newborn neurons of the olfactory bulb (OB). Neural stem- and progenitor cells of the SVZ and SGZ receive dopaminergic input from mesencephalic neurons (Hoglinger et al., 2004, 2014). Dopaminergic neurons of the mesencephalon are located in the substantia nigra pars compacta (SNc) also called A9 region and the ventral tegmental area (VTA) called A10 region (German and Manaye, 1993). While A9 cells predominantly innervate the striatum and the adjacent dorsal SVZ, the ventral SVZ receives dopaminergic input from A10 cells, and the SGZ of the hippocampus is innervated homogenously by A9 and A10 cells (Hoglinger et al., 2014).

A9 and A10 dopaminergic neurons also differ in the expression of Pitx3, a transcription factor with neuroprotective properties (Hwang et al., 2003; Nunes et al., 2003; van den Munckhof et al., 2003). The so called *aphakia* mice lack the expression of Pitx3 due to a homozygote mutation in the Pitx3-gene resulting in a selective degeneration of mesencephalic dopaminergic neurons during embryonic development (Hwang et al., 2003; van den Munckhof et al., 2003; Smidt et al., 2004). This selective degeneration has been used as a genetic animal model of Parkinson's disease in several studies showing typical levodopa responsive motor deficits (Hwang et al., 2005; Le et al., 2015). Moreover, Pitx3 mutant mice also exhibit typical non-motor symptoms of PD like memory dysfunction and depression-related behavior indicating a hippocampal dysfunction (Ardayfio et al., 2008; Kim et al., 2014). As Pitx3 is not expressed in hippocampal neurons the observed cognitive deficits are likely mediated indirectly due to degeneration of dopaminergic projections from the mesencephalon to the striatum and limbic structures. In contrast to toxic or lesion models of PD the *aphakia* mice show a rather selective degeneration of dopaminergic neurons of the SNc (A9-neurons). However, a subset of A10-neurons in the VTA express Pitx3 leading to a partial degeneration of A10 cells due to Pitx3 loss of function (van den Munckhof et al., 2003; Luk et al., 2013). Although, embryonic neurodegeneration as observed in *aphakia* mice does not reflect the natural course of Parkinson's disease, this genetic model has major advantages over toxic or lesion models. One is that these interventional models rather unselectively degenerate neuronal networks and are accompanied by (per-)acute reactions that affect the neuronal system irrespective of the dopaminergic degeneration. Therefore, we believe that the *aphakia* mouse is an optimal animal model to investigate how selective dopaminergic degeneration of mainly A9 neurons affects neurogenesis in the adult neurogenic regions (SVZ and SGZ).

Looking at cell proliferation in the SVZ of adult *aphakia* mice, Lennington et al. found no difference compared to C57BL/6 animals that were used as controls (Lennington et al., 2011). Neuronal tracing via intraventricular injection of WGA-FITC revealed that the SVZ receives dopaminergic innervation from ventrolateral VTA neurons that are mainly unaffected in Pitx3-mutant animals. SVZ proliferation was reduced only after additional MPTP-induced degeneration of these VTA neurons (Lennington et al., 2011).

Whether adult hippocampal neurogenesis is affected in *aphakia* mice has not been investigated so far. As one study claimed an ongoing degeneration of VTA neurons in adult *aphakia* mice (van den Munckhof et al., 2003), we hypothesized that adult neurogenesis in the neurogenic regions is affected in the *aphakia* Parkinson model in a regional and temporal (age-dependent) manner. Therefore, we analyzed neuronal precursor cell proliferation in the SVZ and SGZ of postnatal (3 weeks), juvenile (8 weeks) and senescent (42 weeks) homozygote *aphakia* mice and healthy littermates.

## Methods

### Animal and tissue preparation

Two *aphakia* breeder pairs with a C57BL/6 background were purchased from Jackson Laboratory. Homozygous Pitx3-mutant mice (*aphakia*, ak/ak) and healthy controls (wild-type, WT) were obtained by breeding heterozygous (ak/0) mice.

For the proliferation experiments (sacrificed 24 h after the last BrdU-injection) animals were selected as follows: 4 *aphakia* (1 female, 3 male) and 6 wild type mice (3 female, 3 male) at the age of 3 weeks, 7 *aphakia* and 7 wild type (4 female, 3 male in each group; for BrdU-quantification only female animals were taken into account) at the age of 8 weeks and 4 *aphakia* and 6 wild type at the age of 42 weeks (all male). Another set of 8 weeks old female aphakia and wild type mice (*n* = 4 per group) were selected for the “BrdU-survival experiment” (sacrificed 4 weeks after the last BrdU-injection). Animals lived in regular laboratory cages (3–4 animals per cage) in a 12-h light/dark circadian rhythm and had access to food and water *ad libitum*. To investigate running induced neurogenesis a subset of 8 weeks old male *aphakia* and wild type mice (*n* = 3 per group) had access to a running wheel or lived in regular cages (control condition) for 7 days and were sacrificed at the end of the running period. All Animals were weaned at postnatal day 21. Except for breeding animals that were excluded from the experiment, female and male mice were raised separately. All animal experiments were in accordance with German animal protection laws and institutionally approved by the Technische Unviersität Dresden Animal Care and Use Committee and authorized by the government (Regierungspräsidium Dresden. Germany).

Correct differentiation between *aphakia* and healthy littermates for experiments and breeding required genotyping. DNA was extracted out of a small part of the tail with DNeasy kit, Qiagen. PCR analysis was performed with two pairs of primers to avoid binding problems at the repetitive sequences of the promoter region. Primer pair A Forward 5′-ctctccagcctccctcaata-3′; Reverse 5′-tgttaacgatgtggactaatggt-3′binding before and in the deletion, pair B Forward 5′-ctacgccctcctgtcttctg-3′; Reverse 5′-ccaacctgagagaagcccta-3′covers the whole deletion.

For proliferation experiments the animals received 50 mg/kg bodyweight of Bromodeoxyuridine (BrdU, Sigma, St. Louis, USA) in sterile 0.9% NaCl solution once daily for three consecutive days and were sacrificed 24 h after the last BrdU-injection. For the “survival” experiment 8 weeks old animals were sacrificed 4 weeks after the last BrdU-injection at the age of 12 weeks.

At the end of each experiment all mice were deeply anesthetized with ketamine and perfused transcardially with 4% formaldehyde in cold 0.1 M phosphate buffer. After transcardial perfusion, brains were removed from all animals and stored in 4% formaldehyde overnight at 4°C. Following fixation, brains were transferred to 30% sucrose for 72 h. After sucrose saturation, brains were cut in the coronal plane in 40 μm thick sections on a dry-ice-cooled copper block on a sliding microtome (Leica, Bensheim) and stored in a cryoprotectant solution at −20°C.

### Immunohistochemistry

For BrdU detection slices were incubated in 2 N HCl for 30 min at 37°C prior to 30 min blocking with 5% donkey serum and first antibody incubation over night at 4°C. To determine the total number of BrdU^+^ or TH^+^ cells we used the peroxidase method (ABC system, Vectastain, Vector Laboratories) with biotinylated donkey anti-rat or anti-rabbit antibody (1:500, Dianova) and diaminobenzidine (DAB, Sigma) as chromogen. The secondary antibody incubation time was 2 h. DAB-stained sections were mounted on coated microscope slides and cover-slipped with Neo-Mount (Merck, Darmstadt, Germany). For immunohistochemistry including immunofluorescent triple labeling we used the following primary antibodies: goat anti-DCX (1:200; Santa Cruz Biotech.), rat anti-BrdU (1:250, abcam), rabbit anti-Ki67 (1:200; abcam) rabbit anti-TH (1:300, Pel Freez). The conjugated secondary antibodies consisted of donkey anti-rat, anti-goat and anti-rabbit antibodies (Alexa fluor 488, 555, and 647, Invitrogen) at a dilution of 1:500.

### Quantifications

For absolute cell counts series of every six section (240 μm apart) were stained for BrdU or TH with the peroxidase method. Positive BrdU-cells were counted by a blinded investigator throughout the rostrocaudal extent of the granule cell layer and SGZ of the hippocampus and the SVZ. TH^+^ cells were counted in mesencephalon and spatialized to the VTA and SN by anatomical criteria. The optical dissector method was modified as described previously (Kempermann et al., 2003) in that cells appearing in the uppermost focal plane, when focusing into the section, were not counted to avoid overestimation of the absolute cell number. Absolut cell numbers were counted in every six section of the entire structure of interest. In adult mice the sample size (number of sections) was 9 for the SGZ, 11 for the SVZ and 5 for the SN and VTA. To estimate the total number of BrdU and TH-labeled cells the resulting numbers were multiplied by 6.

To differentiate between cell numbers in the ventral and dorsal part of the SVZ, respectively, the SVZ was virtually halved and divided into a ventral and dorsal part.

Proportional analyses of the cell phenotype were performed in stainings of one-of-twelve series of sections from animals of each group. For any combination of cell phenotype (DCX^+^, BrdU^+^) 100 cells within in granule cell layer of each animal were analyzed (400–480 cells per group) in the confocal microscope (Leica SP5, × 40 oil objective) in sequential scanning mode for co-expression. Double labeling was confirmed by z-series of entire nucleus or cell in question.

### Statistical analyses

Statistical significance was determined by two-sided unpaired *t*-test or 2-way analysis of variance (ANOVA) followed by Bonferroni adjusted *post-hoc t*-test as appropriate. Pearson correlation test was used for correlation analyses using SPSS version 23 (IBM SPSS Statistics Inc., Chicago, USA). Statistical significance was defined as *p* < 0.05 (two-sided). All data are expressed as mean values ± SEM.

## Results

### Young and old Pitx-3 mutant mice show substantial loss of A9 and A moderate reduction of A10 dopaminergic neurons

**Figure 1:**
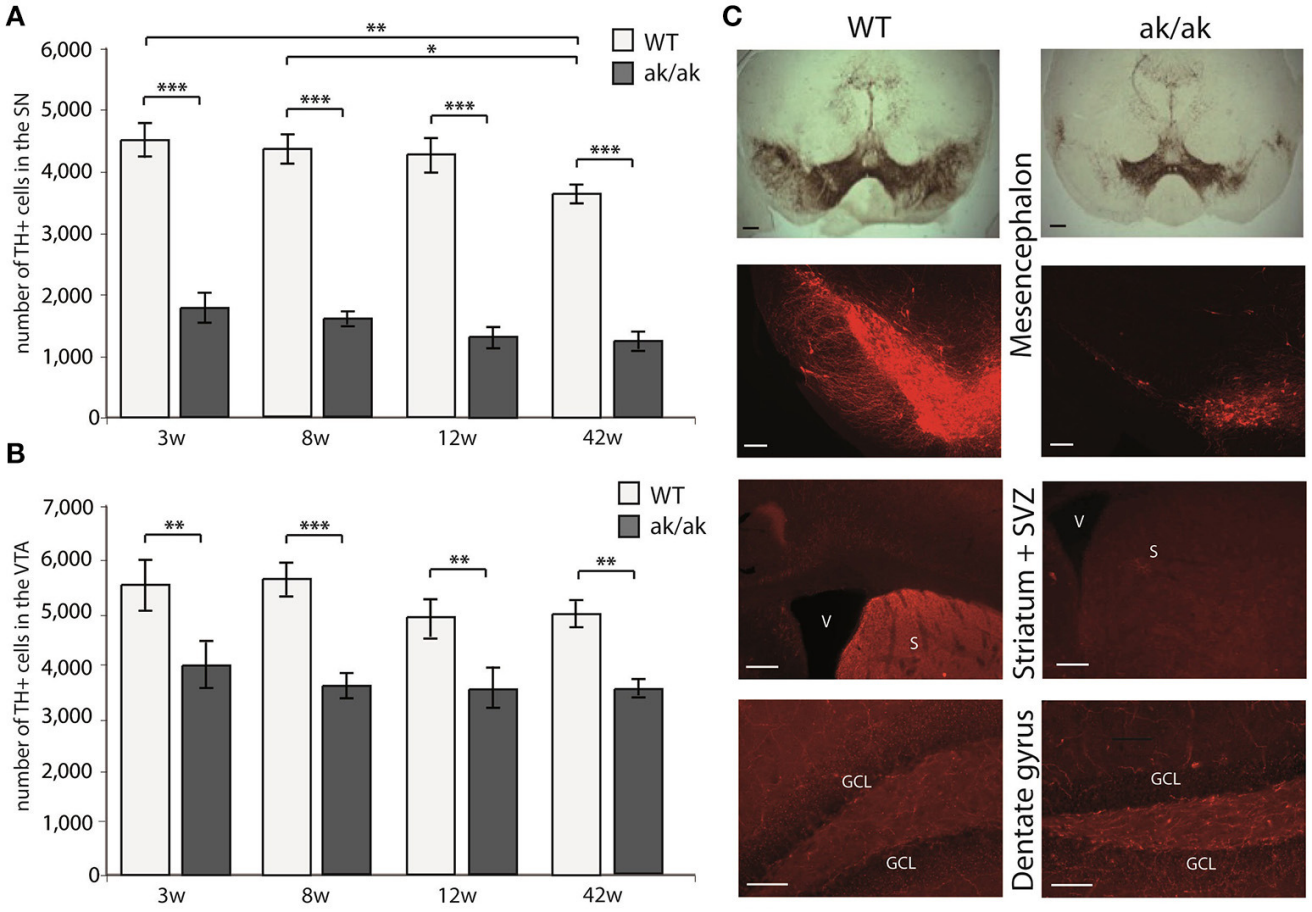
Number of TH^+^ cells in the SN **(A)** and VTA **(B)** of *aphakia* (ak/ak) and wild-type mice (WT) at 3 weeks (3w), 8 weeks (8w), 12 weeks (12w), and 42 weeks (42w) of age (bars indicate mean values ± SEM; ^*^p < 0.05; ^**^p < 0.01; ^***^p < 0.001 from Bonferroni-adjusted *post-hoc t*-test; *n* = 4–7). **(C)** TH-staining of the SN (A9) and VTA (A10) in the mesencephalon shows the preferential degeneration of A9 neurons with a consecutive loss of dopaminergic projection to the dorsal striatum and adjacent SVZ in *aphakia* mice, while the faint dopaminergic innervation of the dentate gyrus seems to be unaffected (scale bars = 200 μm).

Pitx-3 expression in the brain is confined to dopaminergic neurons of the mesencephalon. Loss of Pitx-3 function results in degeneration of these neurons during late embryonic and presumably postnatal development (Hwang et al., 2003; Nunes et al., 2003; van den Munckhof et al., 2003). To confirm this degeneration and to test possible and previously described age-related changes of dopaminergic degeneration in *aphakia* and wild-type mice, we determined the absolute number of TH^+^ neurons in the substantia nigra (SN) and the VTA in animals of different ages (3, 8, 12, and 42 weeks). As the Pitx3-mutation only affects A9 and A10 neurons, but not other catecholaminergic TH^+^ neurons including the noradrenergic system (Nunes et al., 2003), changes in TH^+^ cells and fibers reflect dopaminergic depletion. Two-way ANOVA with genotype and age as fixed factors revealed a robust effect of the genotype on the number of TH^+^ neurons in both dopaminergic regions, effects of age only in the SN region, but no interactions of age and genotype (SN: interaction effect: *p* = 0.575, *F* = 0.7; genotype: *p* < 0.001, *F* = 382.0 and age: *p* = 0.008, *F* = 4.7; VTA: interaction effect: *p* = 0.705, *F* = 0.5; genotype: *p* < 0.001, *F* = 53.4 and age: *p* = 0.106, *F* = 2.2). Already at postnatal week 3, *aphakia* mice have two thirds less dopaminergic neurons in the SN and about one third less in the VTA (Figures 1A,B). 42 weeks old wild-type animals showed a significant reduction of TH-positive SN-neurons compared to younger mice. However, we did not find any other age-related changes in the number of TH-positive neurons in both groups, indicating that neuronal degeneration due to Pitx-3 malfunction exclusively takes place during embryonic and possibly early postnatal (P21) development. The selective neuronal degeneration is accompanied by a robust loss of TH-positive terminals in the neurogenic region of the SVZ adjacent to the striatum but there was not such an obvious alteration in catecholaminergic innervation of the dentate gyrus (Figure 1C) likely due to remaining TH^+^ noradrenergic terminals in the hippocampus that originate from the locus coeruleus, which is unaffected in aphakia mice.

### Dopaminergic degeneration in SN and VTA is accompanied by reduced precursor cell proliferation in juvenile but not middle aged mice

**Figure 2:**
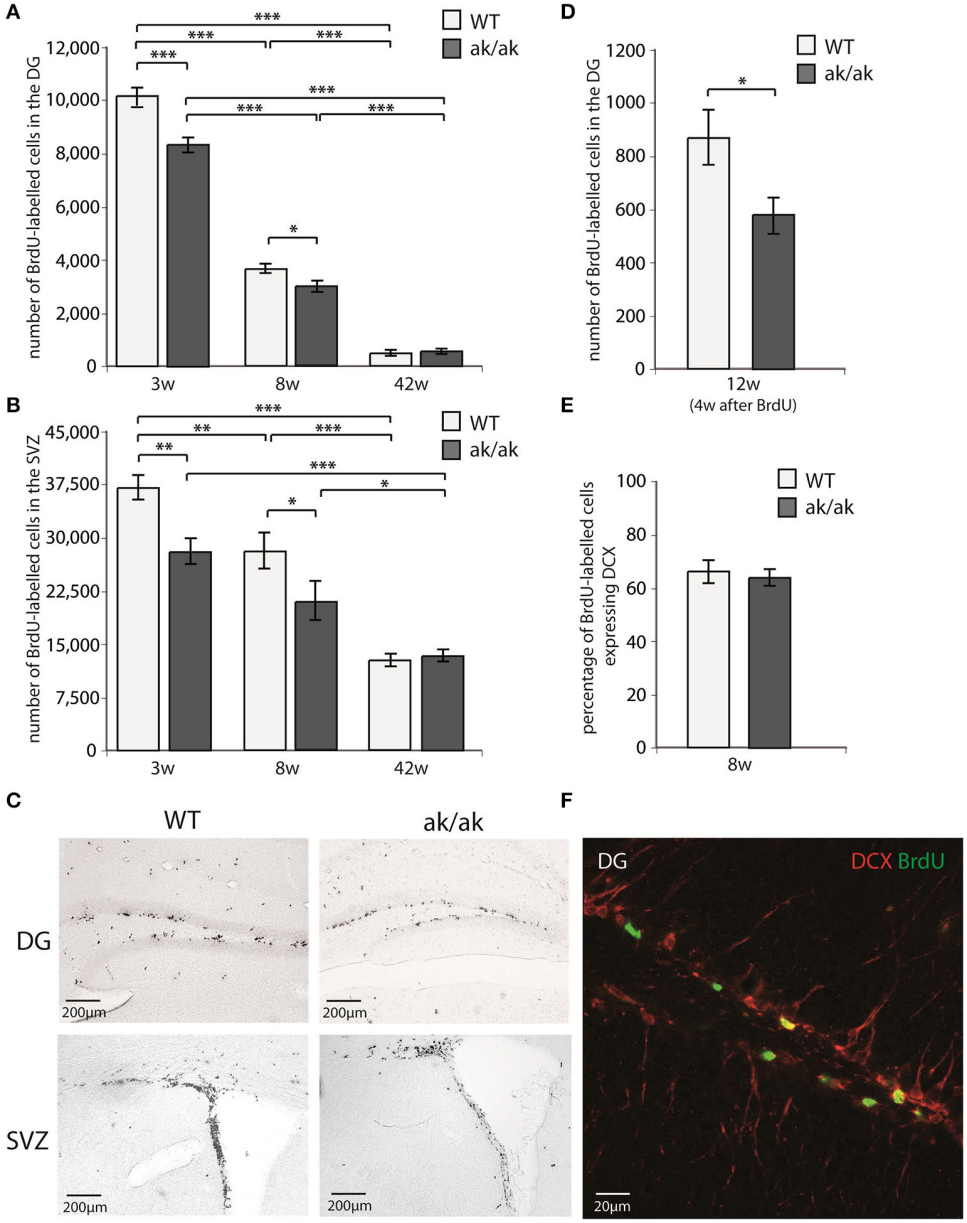
Number of proliferating cells indicated by BrdU-labeling **(A)** in the dentate gyrus (DG) and **(B)** in the subventricular zone (SVZ) of 3 weeks (3w), 8 weeks (8w), and 42 weeks (42w) old *aphakia* (ak/ak) and wild-type mice (WT). **(C)** BrdU-staining of the DG and SVZ of 3 weeks old *aphakia* and WT mice. **(D)** Number BrdU-labeled cells 4 weeks after BrdU-injection in 12 weeks old animals. **(E)** Ratio of BrdU+ cells 1 day after the last BrdU injection expressing neuronal precursor marker DCX. (bars indicate mean values ± SEM; ^*^p < 0.05; ^**^p < 0.01; ^***^p < 0.001 from Bonferroni-adjusted *post-hoc t*-test; *n* = 4–6). **(F)** Representative BrdU/DCX immunofluorescent staining of the denate gyrus.

The number of proliferating cells in neurogenic regions (SGZ and SVZ) of *aphakia* and wild-type mice of different age (3, 8, and 42 weeks) were analyzed using BrdU-labeling. Mice received daily BrdU-injections for 3 days and were sacrificed 24 h after the last BrdU injection. Additionally, the number of dentate gyrus newborn neurons was determined 4 weeks after the last BrdU-injection in 12 weeks old mice (see Figure 2). As expected wild type and *aphakia* mice showed a robust age related decline in the number of proliferating cells, which was more prominent in the SGZ than in the SVZ: Two-way ANOVA with genotype and age as fixed factors for the SGZ revealed that genotype and age have a significant interaction effect on proliferating cell numbers (*p* < 0.001, *F* = 20.3) and significant differences among genotypes (*p* < 0.001, *F* = 35.6) and age groups (*p* < 0.001, *F* = 1267.1). Similar results were obtained for the SVZ: Significant genotype^*^age interaction effect on proliferating cell numbers (*p* = 0.021, *F* = 4.6) and significant differences among genotypes (*p* = 0.001, *F* = 14.7) and age groups (*p* < 0.001, *F* = 61.3). Pair-wise comparison of the two genotypes for each age group revealed that in 3 weeks old *aphakia* mice the number of proliferating BrdU-labeled cells was decreased by 18% in the SGZ (*p* < 0.001, Bonferroni-adjusted *post-hoc t*-test) and 26% in the SVZ (*p* = 0.001) compared to wild-type littermates. At postnatal week 8 we observed a reduction of 18% less proliferating cells in the SGZ of *aphakia* mice (*p* = 0.023) as well as in the SVZ (25%, *p* = 0.017). The impairment of dentate gyrus cell proliferation in 8 weeks old *aphakia* mice results in an even greater decline in the number of newborn cells 4 weeks after the BrdU-injection [33%, *t*_(6)_ = −3.6, *p* = 0.011, Figure 2D]. Co-labeling with neuronal precursor cell marker DCX 1 day after BrdU-injection revealed no changes in the phenotype of proliferating cells (Figure 2E). We found no differences in the number of proliferating cells either in the SGZ (*p* = 0.657) or in the SVZ (*p* = 0.925) of 42 weeks old animals. Taken together, dopamine depletion seems to affect postnatal and juvenile neurogenesis, but not late adult neurogenesis.

Since the dorsal SVZ mainly receives dopaminergic innervation from the SN and the ventral SVZ from VTA neurons, one could expect regional differences in SVZ proliferation in young *aphakia* mice (3 weeks) due to the heterogenic degeneration of VTA and SN neurons. However, we did not find relevant differences in the reduction of BrdU-labeled cells in these subregions [ventral SVZ: −28.4%, *t*_(7)_ = −3.9, *p* < 0.01; dorsal SVZ: −23.2%, *t*_(7)_ = −3.2, *p* < 0.01].

### Amount of cell proliferation in neurogenic regions correlates with the number of SN and VTA dopaminergic neurons in a topographic and age dependent manner

**Figure 3:**
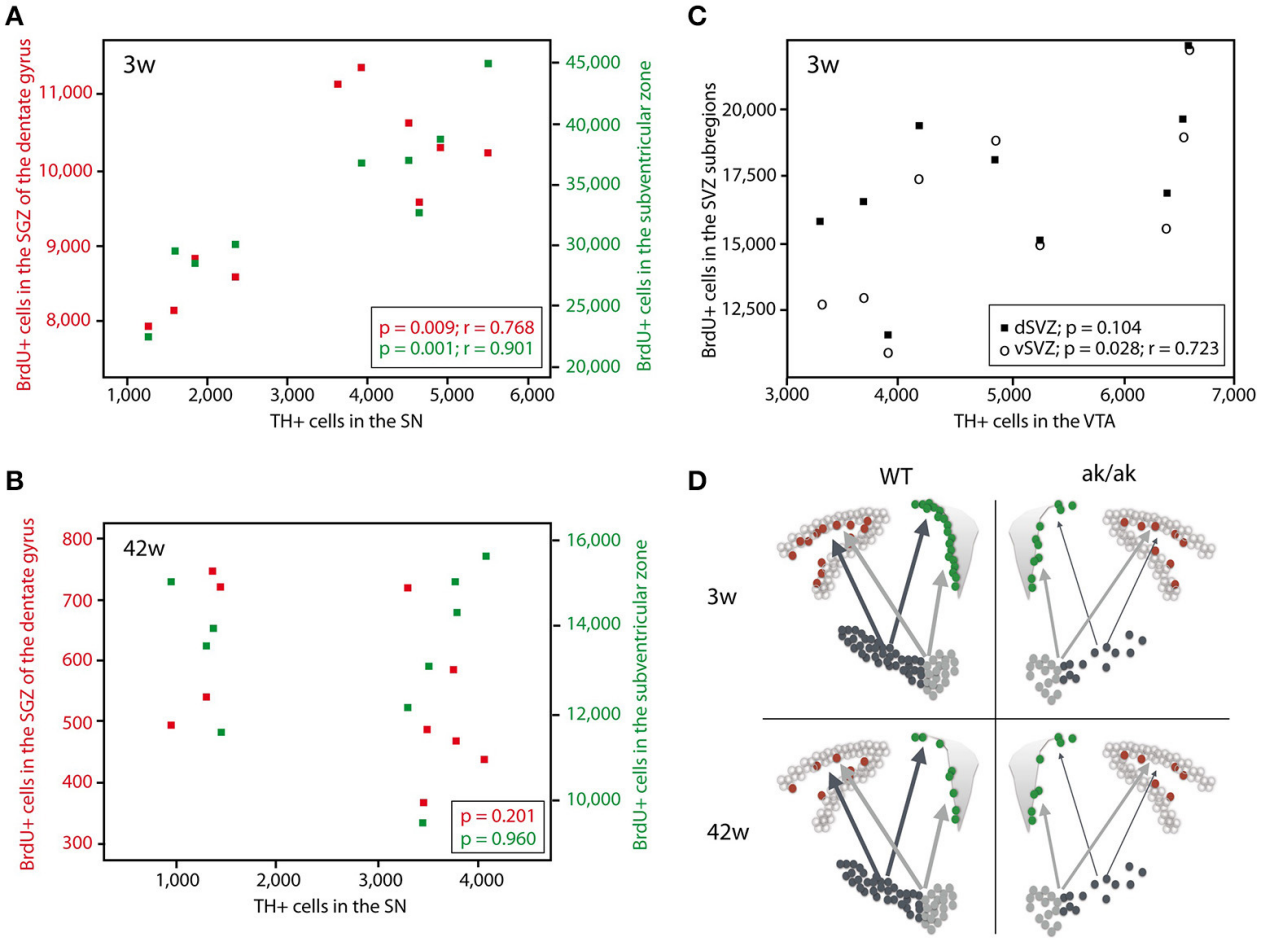
Scatter diagram depicting the number of BrdU-labeled cells in the SGZ (red) and SVZ (green) plotted against the number of TH^+^ cells in the SN in 3 weeks old animals **(A)** and 42 weeks old animals **(B)**. **(C)** Number of BrdU-labeled cells in the dorsal SVZ (black square) and ventral SVZ (circle) plotted against the number of TH^+^ cells in the VTA of 3 weeks old *aphakia* and WT mice. **(D)** Schematic illustration of the degeneration of dopaminergic projection to the neurogenic regions in young and old *aphakia* and WT mice.

To confirm the hypothesis that regional dopamine depletion affects cell proliferation in the neurogenic regions, we looked at the correlation of dopaminergic (TH-positive) neurons and proliferating cells (BrdU-labeled). There is no correlation between the number of VTA dopaminergic neurons and newborn cells in the SGZ, neither in young nor in old animals (data not shown). However, we found a significant correlation of VTA dopaminergic neurons to BrdU-labeled cells in the ventral SVZ (*p* = 0.028, *r* = 0.723) and total SVZ (*p* = 0.049, *r* = 0.669) but not in dorsal SVZ (*p* = 0.104) exclusively in 3 weeks old animals (Figure 3). The number of SN dopaminergic neurons highly correlates with the number of proliferating cells in the SGZ (*p* = 0.009; *r* = 0.768) and SVZ (*p* = 0.001; *r* = 0.901) of 3 weeks old animals but not in the middle aged brain of 42 weeks old *aphakia* and wild type mice (*p* = 0.960 for SVZ; *p* = 0.201 for SGZ). These observations support the assumption that mainly A9-neurons in the SN affect the mitotic activity of adult neural precursor cells only during postnatal development, but not in the aging brain. Thus, loss of VTA neurons that sends dopaminergic projections to the ventral striatum, mainly affects cell proliferation in the ventral SVZ of young animals.

### Stimulation of precursor proliferation by physical activity is not affected in Pitx3-mutant mice

**Figure 4:**
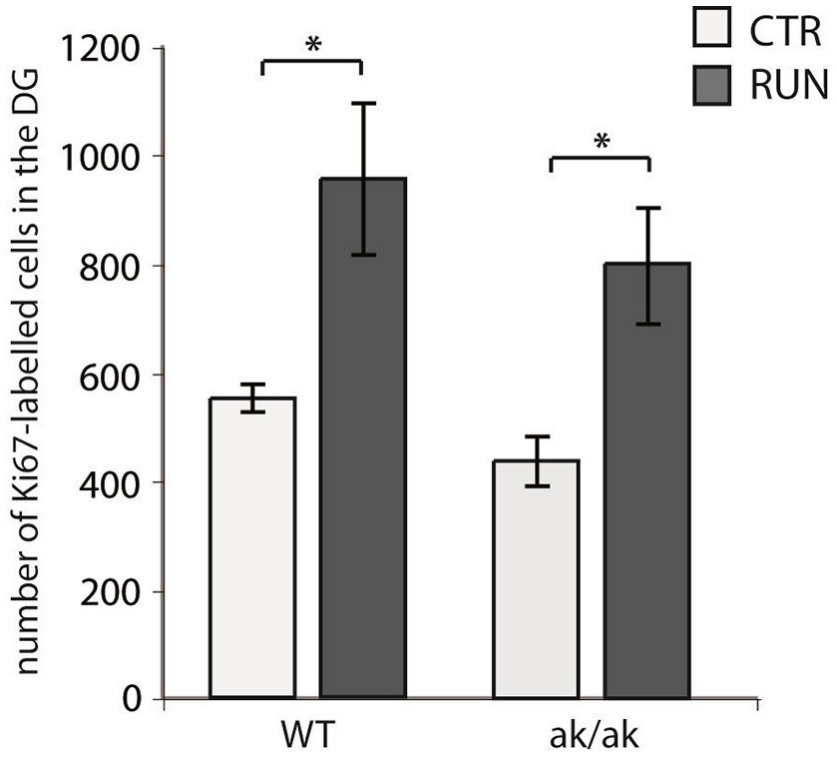
Number of cells in the SGZ of the dentate gyrus expressing the proliferation marker Ki67 in wild-type (WT) and *aphakia* (ak/ak) mice under control conditions (CTR) and after seven days of voluntary exercise in a running wheel (RUN; bars indicate mean values ± SEM). Two-way ANOVA with *post-hoc t*-test and Bonferroni adjustment with genotype and runner group as fixed factors revealed that genotype and runner group have nosignificant interaction effect on Ki67-positive cell numbers (*p* = 0.881, *F* = 0.02) and no significant differences among genotypes (*p* = 0.160, *F* = 2.4), but significant differences between runners and controls (*p* = 0.002, *F* = 19.7); ^*^p < 0.05 from Bonferroni-adjusted *post-hoc t*-test; *n* = 3).

The number of newborn neurons in the adult brain is subject to various behavioral and environmental factors. One of these is physical activity leading to an increased number of proliferating cells, mainly type-2 and 3 cells and stimulates their progression in the precursor lineage in the adult dentate gyrus (van Praag et al., 1999b; Kronenberg et al., 2003; Brandt et al., 2010). The exact mechanisms that are responsible for this pro-neurogenic effect of physical activity are not fully understood (Fischer et al., 2014; Overall et al., 2016). As physical exercise has been shown to alter dopaminergic transmission and to improve cognitive function in animal models of PD as well as PD patients (Tanaka et al., 2009; Petzinger et al., 2013; Klein et al., 2016), we wanted to know whether physical activity also stimulates adult hippocampal neurogenesis in *aphakia* mice that lack the majority of dopaminergic A9 neurons. As described above, at the age of 8 weeks *aphakia* mice show a decreased number of proliferating cells in the SGZ. Giving these mice the opportunity to enhance their physical activity in a running wheel almost doubles the number of SGZ cells expressing the cell cycle marker Ki67 (*p* ≤ 0.05, Figure 4). There was no statistical difference between these *aphakia* and wild type mice after seven days of physical activity indicating that running might rescue the reduced baseline cell proliferation in young *aphakia* mice (Figure 4).

## Discussion

Although, Parkinson's disease (PD) is primarily a movement disorder, most patients also suffer from non-motor symptoms such as depression, cognitive impairment and hyposmia during the natural course of the disease. While the movement disorder is a consequence of nigrostriatal neurodegeneration, it is not clear whether the dopaminergic deficit is also responsible for non-motor symptoms. Structural changes in the hippocampus including reduced cellular plasticity (adult neurogenesis) due to dopamine depletion have been suggested to impair hippocampal function and promote depression and cognitive decline in PD (Hoglinger et al., 2004; Marxreiter et al., 2009; Takamura et al., 2014; Klein et al., 2016; Schlachetzki et al., 2016).

Although, toxic models are widely used as animal models of PD, there are several side effects to consider especially in the context of adult neurogenesis, which represents a very sensitive physiological event. It has been shown that neuronal precursor cells of the SVZ are more vulnerable to MPTP than midbrain dopaminergic cells indicating that dopamine depletion is not the primary reason of impaired adult neurogenesis, at least in the MPTP-model (He et al., 2008). Moreover, toxic or lesion induced dopamine depletion are likely accompanied by acute neuroimmunological reactions that might affect neural stem and precursor cells independently of Parkinson-related degeneration. Therefore, we looked for a non-toxic more specific model, which is characterized by the selective degeneration of SN dopaminergic neurons. *Aphakia* mice carry a mutation in the Pitx3 gene, which also seems to play a role in human susceptibility to PD (Fuchs et al., 2009).

As *aphakia* mice are visually impaired due to microphthalmia and lack of lenses, one could argue that adult neurogenesis alterations might be a consequence of this handicap. Due to the lack of an appropriate control group there are only theoretical considerations that argue against this limitation. In mice, eye-opening occurs about 2 weeks after birth and the development of mature visual cortex activity takes another 1–2 weeks (Rochefort et al., 2009), indicating that relevant differences in visual function between *aphakia* and wild type mice should not occur before 3–4 weeks after birth. However, in our study we found the largest impact on adult precursor cell proliferation in 3 weeks old mice. There was a significant correlation between dopaminergic degeneration and BrdU^+^ cells, but adult neurogenesis alterations did not seem to follow the time course of the development of the visual system. Thus, we suggest that in this animal model, dopaminergic depletion has a much greater regulatory impact on adult neurogenesis than visual impairment.

Previous studies had undoubtedly demonstrated a marked reduction of dopaminergic neurons in the SN of *aphakia* mice due to late embryonic degeneration starting after E13, but presented contradictory results concerning the involvement of the VTA (Hwang et al., 2003; Nunes et al., 2003; van den Munckhof et al., 2003; Smidt et al., 2004). Possible reasons for this divergence might be methodological (e.g., estimation of absolute cell numbers or cell density, retrograde labeling, different anatomical classification and different age of animals). We decided to quantify the absolute cell numbers of TH-positive neurons in the SN and VTA defined by anatomical criteria as early as postnatal day 21 up to 42 weeks old mice. While the majority of TH^+^ fibers in the striatum originate from dopaminergic cells (A9 and A10), the hippocampus also receives TH^+^ noradrenergic projections from the locus coeruleus. However, the Pitx3-mutation in *aphakia* mice remains the locus coeruleus intact (Nunes et al., 2003), thus alterations of hippocampal neurogenesis in *aphakia* mice are independent of the noradrenergic system. Here we show a reduction of TH-cells in the SN of *aphakia* mice to 35% of wild-type mice and 65% of the control-level in the VTA. Except for a slight reduction in SN dopaminergic cells in 42 weeks old wild type mice there was no further age-dependent change in the number of TH-positive cells in both regions. As Pitx3 is not exclusively expressed in SN neurons but also in a subset of VTA neurons, it is likely that the same mechanism that lead to degeneration of A9-neurons in late embryonic development also affects a subset of A10-neurons of the VTA. However, the different extent of neurodegeneration in mesencephalic subregions make the *aphakia* mouse a perfect model to investigate PD and how dopamine depletion might affect adult neurogenesis: (i) The degeneration pattern is very similar to that of PD patients (Smidt et al., 1997; Jellinger, 2001); and (ii) due to the selective degeneration of topographically organized dopaminergic neurons one would expect that the neurogenic regions are unequally affected by the loss of dopaminergic innervation. The majority of dopaminergic projections from the mesencephalon terminate in the striatum, of which the dorsal part is innervated by the SN and the ventral part by the VTA. Accordingly the dorsal and ventral part of the SVZ receives dopaminergic input from the same topographic regions (Lennington et al., 2011; Hoglinger et al., 2014). Both the SN and VTA homogenously innervate the SGZ of the dentate gyrus (Hoglinger et al., 2014). In our experiment we found a reduced cell proliferation in the SVZ of 3 weeks old mice, but no significant differences in the reduction of ventral or dorsal SVZ proliferation. However, the extent of dopaminergic denervation correlates with the degree of precursor cell reduction. Focusing on the number of TH-cells in the VTA (A10) and how this correlates with the amount of BrdU-cells in the dorsal and ventral SVZ at postnatal day 21 revealed a significant correlation only for the ventral part but not the dorsal SVZ. Whereas, reduced proliferation rate in the dorsal SVZ highly correlates with the extent of dopaminergic degeneration of SN neurons (A9). This data underlines how the degeneration of SN and VTA differentially affects precursor cell proliferation in the dorsal and ventral SVZ. As *aphakia* mice show a degeneration of both, A9 and A10 neurons, there might no relevant difference in the reduction of BrdU-cells between ventral and dorsal part of the SVZ, but a region-specific correlation. In contrast, the dentate gyrus receives dopaminergic input from both regions (VTA and SN), but a significant correlation could only be demonstrated for the number of SN dopaminergic neurons and BrdU-cells in the DG, likely due to the more prominent degeneration of the SN compared to the VTA in *aphakia* mice. Further experiments with absolute selective degeneration of A9 and A10 neuron, respectively, are required to clarify the distinct role of dopaminergic projections for adult neurogenic sub-regions.

Interestingly, we found the most distinct reduction of BrdU-labeled cells in the dentate gyrus of young adult animals 4 weeks after BrdU-injection reflecting newborn but already differentiated granule cells. The number of newborn neurons that are functionally integrated into the existing network is regulated by two key events. The first one is cell proliferation, which results in a peak of BrdU-labeled cells during the first 2 days after BrdU injection (Kronenberg et al., 2003; Kempermann et al., 2004; Steiner et al., 2004). The second is the selection of about two thirds of precursor cells and immature neurons during the following days and weeks (Brandt et al., 2003; Kempermann et al., 2004). Proliferation (1 day after last BrdU-injection) in the SGZ of 8 weeks old *aphakia* mice was slightly reduced by 18%, but amount of BrdU^+^ cells 4 weeks after last BrdU-injection decreased by 33%. As a substantial number of BrdU-labeled cells are already postmitotic 1 day after daily BrdU-injection for 3 consecutive days (Brandt et al., 2003, 2012; Encinas et al., 2011), we do not believe that the major reduction during the following 4 weeks is due to decreased proliferation. We rather suggest that dopamine also regulates the selection and survival of newborn neurons. This is in accordance with reports that D1-like receptor activation promotes survival of newborn cells in the adult hippocampus (Takamura et al., 2014).

Although, there is no age dependent change in the dopaminergic degeneration of *aphakia* mice, the initial reduction of cell proliferation in the neurogenic regions in young animals is not further detectable in middle-aged mice. This finding implies that regulatory influence on neurogenic regions by dopaminergic projections diminishes with ageing. A compensatory structural change in *aphakia* mice rescuing dopamine deficit in the SVZ and SGZ are unlikely, because tracing studies in this mouse strain failed to detect any changes in dopaminergic projections to the SVZ (Lennington et al., 2011). We suggest that age-dependent structural changes of the neurogenic niches are responsible for the attenuation of dopaminergic precursor cell regulation in older animals. Hippocampal dopamine receptor density is reduced in aging animals and humans (Amenta et al., 2001; Hemby et al., 2003). Moreover, aging is associated with a reduction of adult neural stem and precursor cells. This age-related decline does not uniformly affect all cell populations, but preferentially transiently amplifying highly proliferative precursor cells, which is the cell source that expresses dopamine receptors and can thereby be stimulated by dopamine (Hoglinger et al., 2004; Lao et al., 2013). Dopaminergic treatment using the dopamine agonist pramipexole increased SGZ cell proliferation in 12 weeks old wild type mice (Salvi et al., 2016), but has no effect on adult hippocampal neurogenesis in 46 weeks old healthy mice (Chiu et al., 2015). Different study designs do not allow a direct comparison of these two observations, but these findings underline the age-dependent sensitivity to dopamine as a regulator of adult precursor cell proliferation. Similar age dependent effects had been found for fluoxetine, a selective serotonin reuptake inhibitor, known to increase adult hippocampal neurogenesis in young animals but fail to do so in elderly mice (Couillard-Despres et al., 2009). As non-motor symptoms has also been shown in *aphakia* mice (Ardayfio et al., 2008), our results indicate that at least in old animals this deficits are not solely attributed to changes in adult neurogenesis.

Physical exercise is a robust stimulator of adult hippocampal precursor proliferation resulting in an increased number of new granule cells and better learning performance in mice (van Praag et al., 1999a,b). Physical training also improves cognitive and motor functions in PD patients (Tanaka et al., 2009; Petzinger et al., 2013). Young *aphakia* mice (8 weeks) that had less proliferating cells in the SGZ of the DG under control conditions doubled the number of proliferating cells after 1 week of physical exercise (voluntary wheel running). Despite of the dramatic depletion of midbrain dopaminergic neurons, these mice respond to the stimulus in the same way as wild-type mice. Thus, running induced cell proliferation seems to be independent of dopaminergic degeneration, which has further implications for adjuvant non-pharmacological treatment strategies of non-motor symptoms in PD.

Together, the Pitx3-mutant animal model of PD is characterized by a moderate reduction of precursor cell proliferation in the SGZ and SVZ only in young animals (3–8 weeks) while precursor cell proliferation in older animals and stimulation of hippocampal neurogenesis remain unaffected. Although, this genetic animal model does not reflect the natural course of PD, as the dopaminergic depletion already starts during embryonic development, it gives us the opportunity to investigate adult neurogenesis after PD-like selective degeneration of the nigrostriatal and—to a lesser degree—the mesolimbic system and independently of any toxic or lesion induced side effects. Recent studies using genetic animal models of PD (like α-synuclein overexpression) confirmed an impairment of adult neurogenesis (Marxreiter et al., 2009, 2013a; Kohl et al., 2016; Schlachetzki et al., 2016). Whether α-synuclein expression is directly involved in this impairment or rather reduces neurogenesis as a consequence of dopaminergic or even serotonergic degeneration is still being debated. However, most of these studies investigated adult neurogenesis in young (around 8 weeks old) animals, which we suggest are more susceptible to dopaminergic depletion than the brains of older animals. As PD mainly affects the senescent brain, future studies should take into account that adult neurogenesis in the aging brain might behave differently.

## Author contributions

MB, DK, AH, AM, KK, and AS conceived and designed the experiments. MB, DK, AH, and AM performed the experiments. MB, DK, AM, and AS analyzed the data. MB, DK, AH, AM, KS, and AS wrote and edited the manuscript.

### Conflict of interest statement

The authors declare that the research was conducted in the absence of any commercial or financial relationships that could be construed as a potential conflict of interest.

## References

[B1] Amenta F. Mignini F. Ricci A. Sabbatini M. Tomassoni D. Tayebati S. K. (2001). Age-related changes of dopamine receptors in the rat hippocampus: a light microscope autoradiography study. Mech. Ageing Dev. 122, 2071–2083. 10.1016/S0047-6374(01)00317-7 11589924

[B2] Ardayfio P. Moon J. Leung K. K. Youn-Hwang D. Kim K. S. (2008). Impaired learning and memory in Pitx3 deficient aphakia mice: a genetic model for striatum-dependent cognitive symptoms in Parkinson's disease. Neurobiol. Dis. 31, 406–412. 10.1016/j.nbd.2008.05.017 18573342 PMC2594011

[B3] Brandt M. D. Hubner M. Storch A. (2012). Brief report: adult hippocampal precursor cells shorten S-phase and total cell cycle length during neuronal differentiation. Stem Cells 30, 2843–2847. 10.1002/stem.1244 22987479

[B4] Brandt M. D. Jessberger S. Steiner B. Kronenberg G. Reuter K. Bick-Sander A.. (2003). Transient calretinin expression defines early postmitotic step of neuronal differentiation in adult hippocampal neurogenesis of mice. Mol. Cell Neurosci. 24, 603–613. 10.1016/S1044-7431(03)00207-0 14664811

[B5] Brandt M. D. Maass A. Kempermann G. Storch A. (2010). Physical exercise increases Notch activity, proliferation and cell cycle exit of type-3 progenitor cells in adult hippocampal neurogenesis. Eur. J. Neurosci. 32, 1256–1264. 10.1111/j.1460-9568.2010.07410.x 20950279

[B6] Chiu W. H. Depboylu C. Hermanns G. Maurer L. Windolph A. Oertel W. H.. (2015). Long-term treatment with L-DOPA or pramipexole affects adult neurogenesis and corresponding non-motor behavior in a mouse model of Parkinson's disease. Neuropharmacology 95, 367–376. 10.1016/j.neuropharm.2015.03.020 25839898

[B7] Couillard-Despres S. Wuertinger C. Kandasamy M. Caioni M. Stadler K. Aigner R.. (2009). Ageing abolishes the effects of fluoxetine on neurogenesis. Mol. Psychiatry 14, 856–864. 10.1038/mp.2008.147 19139747

[B8] Diaz J. Ridray S. Mignon V. Griffon N. Schwartz J. C. Sokoloff P. (1997). Selective expression of dopamine D3 receptor mRNA in proliferative zones during embryonic development of the rat brain. J. Neurosci. 17, 4282–4292. 9151745 10.1523/JNEUROSCI.17-11-04282.1997 PMC6573556

[B9] Encinas J. M. Michurina T. V. Peunova N. Park J. H. Tordo J. Peterson D. A.. (2011). Division-coupled astrocytic differentiation and age-related depletion of neural stem cells in the adult hippocampus. Cell Stem Cell 8, 566–579. 10.1016/j.stem.2011.03.010 21549330 PMC3286186

[B10] Feng R. Rampon C. Tang Y. P. Shrom D. Jin J. Kyin M.. (2001). Deficient neurogenesis in forebrain-specific presenilin-1 knockout mice is associated with reduced clearance of hippocampal memory traces. Neuron 32, 911–926. 10.1016/S0896-6273(01)00523-2 11738035

[B11] Fischer T. J. Walker T. L. Overall R. W. Brandt M. D. Kempermann G. (2014). Acute effects of wheel running on adult hippocampal precursor cells in mice are not caused by changes in cell cycle length or S phase length. Front. Neurosci. 8:314. 10.3389/fnins.2014.00314 25339861 PMC4186268

[B12] Fuchs J. Mueller J. C. Lichtner P. Schulte C. Munz M. Berg D.. (2009). The transcription factor PITX3 is associated with sporadic Parkinson's disease. Neurobiol. Aging 30, 731–738. 10.1016/j.neurobiolaging.2007.08.014 17905480

[B13] German D. C. Manaye K. F. (1993). Midbrain dopaminergic neurons (nuclei A8, A9, and A10): three-dimensional reconstruction in the rat. J. Comp. Neurol. 331, 297–309. 10.1002/cne.903310302 8514911

[B14] He X. J. Yamauchi H. Uetsuka K. Nakayama H. (2008). Neurotoxicity of MPTP to migrating neuroblasts: studies in acute and subacute mouse models of Parkinson's disease. Neurotoxicology 29, 413–420. 10.1016/j.neuro.2008.02.007 18387672

[B15] Hemby S. E. Trojanowski J. Q. Ginsberg S. D. (2003). Neuron-specific age-related decreases in dopamine receptor subtype mRNAs. J. Comp. Neurol. 456, 176–183. 10.1002/cne.10525 12509874 PMC4048549

[B16] Hill A. S. Sahay A. Hen R. (2015). Increasing Adult Hippocampal neurogenesis is sufficient to reduce anxiety and depression-like behaviors. Neuropsychopharmacology 40, 2368–2378. 10.1038/npp.2015.85 25833129 PMC4538351

[B17] Hoglinger G. U. Arias-Carrion O. Ipach B. Oertel W. H. (2014). Origin of the dopaminergic innervation of adult neurogenic areas. J. Comp. Neurol. 522, 2336–2348. 10.1002/cne.23537 24424877

[B18] Hoglinger G. U. Rizk P. Muriel M. P. Duyckaerts C. Oertel W. H. Caille I.. (2004). Dopamine depletion impairs precursor cell proliferation in Parkinson disease. Nat. Neurosci. 7, 726–735. 10.1038/nn1265 15195095

[B19] Hwang D. Y. Ardayfio P. Kang U. J. Semina E. V. Kim K. S. (2003). Selective loss of dopaminergic neurons in the substantia nigra of Pitx3-deficient aphakia mice. Brain Res. Mol. Brain Res. 114, 123–131. 10.1016/S0169-328X(03)00162-1 12829322

[B20] Hwang D. Y. Fleming S. M. Ardayfio P. Moran-Gates T. Kim H. Tarazi F. I.. (2005). 3,4-dihydroxyphenylalanine reverses the motor deficits in Pitx3-deficient aphakia mice: behavioral characterization of a novel genetic model of Parkinson's disease. J. Neurosci. 25, 2132–2137. 10.1523/JNEUROSCI.3718-04.2005 15728853 PMC6726071

[B21] Jellinger K. A. (2001). The pathology of Parkinson's disease. Adv. Neurol. 86, 55–72. 11554010

[B22] Kempermann G. Kronenberg G. (2003). Depressed new neurons–adult hippocampal neurogenesis and a cellular plasticity hypothesis of major depression. Biol. Psychiatry 54, 499–503. 10.1016/S0006-3223(03)00319-6 12946878

[B23] Kempermann G. Gast D. Kronenberg G. Yamaguchi M. Gage F. H. (2003). Early determination and long-term persistence of adult-generated new neurons in the hippocampus of mice. Development 130, 391–399. 10.1242/dev.00203 12466205

[B24] Kempermann G. Jessberger S. Steiner B. Kronenberg G. (2004). Milestones of neuronal development in the adult hippocampus. Trends Neurosci. 27, 447–452. 10.1016/j.tins.2004.05.013 15271491

[B25] Kim K. S. Kang Y. M. Kang Y. Park T. S. Park H. Y. Kim Y. J.. (2014). Pitx3 deficient mice as a genetic animal model of co-morbid depressive disorder and parkinsonism. Brain Res. 1552, 72–81. 10.1016/j.brainres.2014.01.023 24480473

[B26] Klein C. Rasinska J. Empl L. Sparenberg M. Poshtiban A. Hain E. G.. (2016). Physical exercise counteracts MPTP-induced changes in neural precursor cell proliferation in the hippocampus and restores spatial learning but not memory performance in the water maze. Behav. Brain Res. 307, 227–238. 10.1016/j.bbr.2016.02.040 27012392

[B27] Kohl Z. Ben Abdallah N. Vogelgsang J. Tischer L. Deusser J. Amato D.. (2016). Severely impaired hippocampal neurogenesis associates with an early serotonergic deficit in a BAC alpha-synuclein transgenic rat model of Parkinson's disease. Neurobiol. Dis. 85, 206–217. 10.1016/j.nbd.2015.10.021 26523794 PMC4974940

[B28] Kronenberg G. Reuter K. Steiner B. Brandt M. D. Jessberger S. Yamaguchi M.. (2003). Subpopulations of proliferating cells of the adult hippocampus respond differently to physiologic neurogenic stimuli. J. Comp. Neurol. 467, 455–463. 10.1002/cne.10945 14624480

[B29] Lao C. L. Lu C. S. Chen J. C. (2013). Dopamine D3 receptor activation promotes neural stem/progenitor cell proliferation through AKT and ERK1/2 pathways and expands type-B and -C cells in adult subventricular zone. Glia 61, 475–489. 10.1002/glia.22449 23322492

[B30] Le W. Zhang L. Xie W. Li S. Dani J. A. (2015). Pitx3 deficiency produces decreased dopamine signaling and induces motor deficits in Pitx3(-/-) mice. Neurobiol. Aging 36, 3314–3320. 10.1016/j.neurobiolaging.2015.08.012 26363812 PMC4641756

[B31] Lennington J. B. Pope S. Goodheart A. E. Drozdowicz L. Daniels S. B. Salamone J. D.. (2011). Midbrain dopamine neurons associated with reward processing innervate the neurogenic subventricular zone. J. Neurosci. 31, 13078–13087. 10.1523/JNEUROSCI.1197-11.2011 21917791 PMC3180856

[B32] Luk K. C. Rymar V. V. van den Munckhof P. Nicolau S. Steriade C. Bifsha P.. (2013). The transcription factor Pitx3 is expressed selectively in midbrain dopaminergic neurons susceptible to neurodegenerative stress. J. Neurochem. 125, 932–943. 10.1111/jnc.12160 23331067

[B33] Marxreiter F. Ettle B. May V. E. Esmer H. Patrick C. Kragh C. L.. (2013a). Glial A30P alpha-synuclein pathology segregates neurogenesis from anxiety-related behavior in conditional transgenic mice. Neurobiol. Dis. 59, 38–51. 10.1016/j.nbd.2013.07.004 23867236 PMC4324756

[B34] Marxreiter F. Nuber S. Kandasamy M. Klucken J. Aigner R. Burgmayer R.. (2009). Changes in adult olfactory bulb neurogenesis in mice expressing the A30P mutant form of alpha-synuclein. Eur. J. Neurosci. 29, 879–890. 10.1111/j.1460-9568.2009.06641.x 19291219

[B35] Marxreiter F. Regensburger M. Winkler J. (2013b). Adult neurogenesis in Parkinson's disease. Cell Mol. Life Sci. 70, 459–473. 10.1007/s00018-012-1062-x 22766974 PMC11113680

[B36] Nunes I. Tovmasian L. T. Silva R. M. Burke R. E. Goff S. P. (2003). Pitx3 is required for development of substantia nigra dopaminergic neurons. Proc. Natl. Acad. Sci. U.S.A. 100, 4245–4250. 10.1073/pnas.0230529100 12655058 PMC153078

[B37] Overall R. W. Walker T. L. Fischer T. J. Brandt M. D. Kempermann G. (2016). Different mechanisms must be considered to explain the increase in hippocampal neural precursor cell proliferation by physical activity. Front. Neurosci. 10:362. 10.3389/fnins.2016.00362 27536215 PMC4971098

[B38] Petzinger G. M. Fisher B. E. McEwen S. Beeler J. A. Walsh J. P. Jakowec M. W. (2013). Exercise-enhanced neuroplasticity targeting motor and cognitive circuitry in Parkinson's disease. Lancet Neurol. 12, 716–726. 10.1016/S1474-4422(13)70123-6 23769598 PMC3690528

[B39] Reichmann H. Brandt M. D. Klingelhoefer L. (2016). The nonmotor features of Parkinson's disease: pathophysiology and management advances. Curr. Opin. Neurol. 29, 467–473. 10.1097/WCO.0000000000000348 27262147

[B40] Rochefort N. L. Garaschuk O. Milos R. I. Narushima M. Marandi N. Pichler B.. (2009). Sparsification of neuronal activity in the visual cortex at eye-opening. Proc. Natl. Acad. Sci. U.S.A. 106, 15049–15054. 10.1073/pnas.0907660106 19706480 PMC2736444

[B41] Salvi R. Steigleder T. Schlachetzki J. C. Waldmann E. Schwab S. Winner B.. (2016). Distinct effects of chronic dopaminergic stimulation on hippocampal neurogenesis and striatal doublecortin expression in adult mice. Front. Neurosci. 10:77. 10.3389/fnins.2016.00077 27013940 PMC4786557

[B42] Schlachetzki J. C. Grimm T. Schlachetzki Z. Ben Abdallah N. M. Ettle B. Vohringer P.. (2016). Dopaminergic lesioning impairs adult hippocampal neurogenesis by distinct modification of alpha-synuclein. J. Neurosci. Res. 94, 62–73. 10.1002/jnr.23677 26451750

[B43] Schoenfeld T. J. Cameron H. A. (2015). Adult neurogenesis and mental illness. Neuropsychopharmacology 40, 113–128. 10.1038/npp.2014.230 25178407 PMC4262910

[B44] Shors T. J. Miesegaes G. Beylin A. Zhao M. Rydel T. Gould E. (2001). Neurogenesis in the adult is involved in the formation of trace memories. Nature 410, 372–376. 10.1038/35066584 11268214

[B45] Smidt M. P. Smits S. M. Bouwmeester H. Hamers F. P. van der Linden A. J. Hellemons A. J.. (2004). Early developmental failure of substantia nigra dopamine neurons in mice lacking the homeodomain gene Pitx3. Development 131, 1145–1155. 10.1242/dev.01022 14973278

[B46] Smidt M. P. van Schaick H. S. Lanctot C. Tremblay J. J. Cox J. J. van der Kleij A. A.. (1997). A homeodomain gene Ptx3 has highly restricted brain expression in mesencephalic dopaminergic neurons. Proc. Natl. Acad. Sci. U.S.A. 94, 13305–13310. 10.1073/pnas.94.24.13305 9371841 PMC24304

[B47] Snyder J. S. Soumier A. Brewer M. Pickel J. Cameron H. A. (2011). Adult hippocampal neurogenesis buffers stress responses and depressive behaviour. Nature 476, 458–461. 10.1038/nature10287 21814201 PMC3162077

[B48] Steiner B. Kronenberg G. Jessberger S. Brandt M. D. Reuter K. Kempermann G. (2004). Differential regulation of gliogenesis in the context of adult hippocampal neurogenesis in mice. Glia 46, 41–52. 10.1002/glia.10337 14999812

[B49] Takamura N. Nakagawa S. Masuda T. Boku S. Kato A. Song N.. (2014). The effect of dopamine on adult hippocampal neurogenesis. Prog. Neuropsychopharmacol. Biol. Psychiatry 50, 116–124. 10.1016/j.pnpbp.2013.12.011 24374069

[B50] Tanaka K. Quadros A. C. Jr. Santos R. F. Stella F. Gobbi L. T. Gobbi S. (2009). Benefits of physical exercise on executive functions in older people with Parkinson's disease. Brain Cogn. 69, 435–441. 10.1016/j.bandc.2008.09.008 19006643

[B51] van den Munckhof P. Luk K. C. Ste-Marie L. Montgomery J. Blanchet P. J. Sadikot A. F.. (2003). Pitx3 is required for motor activity and for survival of a subset of midbrain dopaminergic neurons. Development 130, 2535–2542. 10.1242/dev.00464 12702666

[B52] van Praag H. Christie B. R. Sejnowski T. J. Gage F. H. (1999a). Running enhances neurogenesis, learning, and long-term potentiation in mice. Proc. Natl. Acad. Sci. U.S.A. 96, 13427–13431. 10.1073/pnas.96.23.13427 10557337 PMC23964

[B53] van Praag H. Kempermann G. Gage F. H. (1999b). Running increases cell proliferation and neurogenesis in the adult mouse dentate gyrus. Nat. Neurosci. 2, 266–270. 10.1038/6368 10195220

